# Increased Yearling Weight Gain Is Associated with a Distinct Faecal Microbial Profile

**DOI:** 10.3390/ani13193062

**Published:** 2023-09-29

**Authors:** Brianna N. Maslen, Christian Duff, Samuel A. Clark, Julius Van der Werf, Jason D. White, Sameer D. Pant

**Affiliations:** 1 Gulbali Institute, Charles Sturt University, Boorooma Street, Wagga Wagga, NSW 2678, Australia; bmaslen@csu.edu.au; 2 Angus Australia, Armidale, NSW 2350, Australia; christian@angusaustralia.com.au; 3 School of Environmental and Rural Science, University of New England, Armidale, NSW 2350, Australia; sam.clark@une.edu.au (S.A.C.); jvanderw@une.edu.au (J.V.d.W.); 4 Research Office, Charles Sturt University, Wagga Wagga, NSW 2650, Australia; jwhite@csu.edu.au

**Keywords:** microbial profiles, average daily gain, 16S rRNA, weaning weight, yearling weight, Angus steers, faecal

## Abstract

**Simple Summary:**

Gut microbial profiles have been shown to influence a range of physiological processes contributing to animal performance. In particular, gut microbiota have a close relationship with digestion and metabolism, indicating they may be correlated to weight gain. As previous studies have discussed, gut microbiota ranging from the rumen to the hindgut can influence and potentially indicate differences in important production traits such as milk composition in dairy cattle and feed efficiency in beef cattle. Therefore, the objective of this study was to characterise the faecal microbial profiles of Angus steers with high and low average daily gain (ADG) at both weaning and yearling stages. The results indicate that at yearling stage, there was a distinct difference in the faecal microbial profiles of steers with high or low ADG. Primarily, we observed a reduction in species richness and decrease in between-sample phylogenetic similarity in the high ADG group.

**Abstract:**

Microbial communities inhabiting the gut have the ability to influence physiological processes contributing to livestock production and performance. Livestock enterprises rely on animal production traits such as growth performance for profit. Previous studies have shown that gut microbiota are correlated to growth performance and could even influence it. The aim of this study was to characterise the faecal microbial profiles of Angus steers with high and low ADG at both weaning and yearling stages by profiling 16S rRNA gene sequences from rectal faecal samples. When microbial profiles were compared in terms of relative abundances, LEfSe analysis, alpha diversity metrics, and beta diversity, at the weaning stage, few significant differences were found between the high and low ADG groups. However, at yearling stage, microbial profiles significantly differed between the high and low ADG groups. The relative abundances of eight phyla and six genera significantly differed between the two groups. Alpha diversity metrics showed a significant decrease (*p* = 0.001) in species richness in the high ADG group. Similarly, beta diversity analysis showed that samples clustered clearly according to high and low ADG groups at yearling stage, indicating that phylogenetic similarity between the two ADG groups was significantly reduced (*p* = 0.005).

## 1. Introduction

Growth performance is a crucial determinant of the profitability of livestock enterprises [1] that can be influenced by a range of factors such as feed intake and feed efficiency. Previous studies have found that growth performance in livestock, along with some of the underlying factors that influence growth performance, can be influenced by gut microorganisms [2,3,4]. Microbial communities, referred to as microbiota, modulate a wide range of physiological processes and contribute to the overall health of the host [5,6]. Specifically, in the gastrointestinal tract, studies have shown that microbiota potentially influence host energy homeostasis, digestion, and metabolism [5,6,7,8]. Therefore, it is reasonable to hypothesise that gut microbiota may contribute to the growth performance of livestock.

Murine and bovine studies have shown that gut microorganisms can aid digestion by increasing the energy extracted from food and also regulate fat storage [2]. A murine study showed that the gut microbiota in mice selected for obesity were more efficient in harvesting and storing dietary energy than in non-obese mice [3]. Furthermore, if these ‘obese microbiota’ were transferred to lean mice using faecal matter transplants, they resulted in a rapid increase in the total body fat content [3]. The overall community structure of gut microbiota showed a greater relative abundance of Firmicutes that was correlated to the obese donor when compared to the lean donor [3]. Specifically in dairy cattle, the ruminal microbiota was found to influence milk composition and feed efficiency, with higher proportions of Firmicutes being strongly correlated with higher daily milk-fat yield, which could be a result of better feed efficiency [4]. This suggests microorganisms can indirectly control energy storage and, therefore, feed efficiency, and thus they could be used in the future to predict growth performance between subjects [2]. 

Average daily gain (ADG) is often used in the livestock industry to track the growth of meat animals. Studies in weight gain in livestock use ADG as a simple and effective measure to determine the performance of individual animals. Along with feed efficiency and feed intake, ADG can also be influenced by microorganisms within the gut. A study conducted on steers found that microorganisms in the rumen differed significantly in steers with differing feed efficiency [9]. When ADG was looked at individually in comparison to feed efficiency, the same microorganisms correlating to high or low feed efficiencies were identified. Additionally, the same microbial abundance trends were observed when comparing feed efficiency to ADG as a growth indicator [9]. Evidence suggests that microorganisms influence feed efficiency and ADG in the same way. The Firmicutes-to-Bacteroidetes ratio has been found to be correlated with average daily gain in cattle [9]. In the rumen, when Firmicutes increased (or Bacteroidetes decreased), ADG increased [9]. These results indicate that the quantitative or qualitative presence of specific group of microorganisms in the gut can affect the energy-harvesting capacity of cattle and could be used as an indicator for different weight states in individuals.

Understanding the relationship between gut microbiota profiles and growth in beef cattle can offer new insights into mechanisms underlying production traits such as growth rates. Microorganisms and their role in growth have been well documented in a range of species [3,4,10]. Therefore, the aim of this study was to characterise the faecal microbiota profiles in cattle with both high and low growth rates at both weaning and yearling stage in order to determine whether there are any significant differences between the two groups at the two life stages. This presents further opportunities to understand the correlation microbial profiles may have with animal growth performance and in the future.

## 2. Materials and Methods

Animal experiments were conducted at Charles Sturt University (CSU) in Wagga Wagga, New South Wales (NSW), Australia. The experimental protocols were reviewed and approved by Charles Sturt University’s Animal Care and Ethics Committee (reference number: A20347).

### 2.1. Animal Husbandry, Experimental Design, and Sampling of Cattle

A total of 74 steers, raised on CSU’s commercial farm in Wagga Wagga, NSW, Australia, were sampled for this study. Perennial pasture, consisting of Phalaris and clover, was available ad libitum to cattle from calving to weaning. During weaning, Lucerne silage was offered over a 14 day period. After weaning, cattle were grazed on pasture consisting of Fescue, clover, and Lucerne. Rectal faecal samples and weights were collected at ≈6 months of age (weaning stage) and again at ≈12 months of age (yearling stage) during the routine weigh-ins. Immediately after collection, faecal samples were stored in dry ice and transported to the laboratory where they were kept at −20 °C. From the total steers sampled (*n* = 74), 60 steers that had faecal samples and weight data collected at both weaning and yearling stages were included in this study. These rectal faecal samples underwent DNA extraction and subsequent 16S rRNA gene sequencing.

### 2.2. Measurement of Average Daily Gain

The ADG of cattle was calculated by weighing cattle at ≈6 months of age (weaners) and again at ≈12 months of age (yearlings) and dividing the difference by the total number of days between weaning and yearling stages. From the 60 cattle chosen for sequencing, ADG was used to rank them from high growth performance to low growth performance. The top ranked 20 animals (high ADG) and bottom ranked 20 animals (low ADG) were then selected for further analyses. ADG was utilised, as the data from individual steers was required rather than the average of the group. 

### 2.3. DNA Extractions of Faecal Samples and 16S rRNA Gene Sequencing

Genomic microbial DNA was extracted from faecal samples using the Quick-DNA™ Fecal/Soil Microbe Miniprep Kit (Zymo Research, Irvine, CA, USA) as per the manufacturer’s instructions. Final yield and quality of extracted DNA were determined via a NanoDrop 2000 Spectrophotometer (Thermo Scientific, Scoresby, VIC, Australia), and subsequently, samples were subjected to 16S rRNA gene sequencing at the Novogene sequencing facility in Singapore (Singapore). The amplified regions V3–V4 were used to construct a fragment library. Paired-end sequencing was then performed on this library based on the Illumina NovaSeq sequencing platform using the primer set of 341F (5′-CCTAYGGGRBGCASCAG-3′)—806R (5′-GGACTACNNGGGTATCTAAT-3′). All 120 samples passed quality control at the Novogene sequencing facility. 

### 2.4. Sequencing Data and Statistical Analysis

Raw data were spliced and filtered to obtain clean data. DADA2 [11] was used, based on the clean data, to reduce noise and filter out the sequences with abundances less than 5. This produced the final amplicon sequence variants (ASVs). Quantitative Insights Into Microbial Ecology (QIIME2) [12] was used to annotate the species of each ASV, and these were used to interpret species information and abundance distribution. Relative abundances (average and individual comparisons), alpha diversity analysis based on operational taxonomic units (OTU’s) and Chao1, and beta diversity analysis on unweighted UniFrac distances (PCoA) were subsequently carried out using ASV’s annotated by QIIME2. Based on the results of species annotations at the phylum and genus levels, a bar chart of taxa relative abundances was generated using R Studio (Version: 2022.07.2) [13] and packages reshape2, ggplot2, and ggpubr [14,15,16]. To further explore the relative abundances of ASVs, a multivariate analysis of variance (MANOVA) was carried out in R Studio. The top 15 most abundant phyla and top 20 most abundant genera were used to identify the statistical significance of individual taxa between the two groups (*p* = 0.05). Alpha diversity metrics were analysed to infer species richness (within-sample phylogenetic similarities). The OTUs and Chao1 diversity measures were displayed using R Studio (packages XML, ggplot2, matrixStats, plyr, and ggpubr) [15,16,17,18,19]. A Krustal–Wallis pairwise significance test was used to analyse whether the differences in diversity between groups were significant. Principal coordinate analysis (PCoA) was performed to explore the differences in community structures between different groups (between-sample phylogenetic similarities). Principal coordinates from unweighted UniFrac distances were used to visualise the multidimensional data, where the maximum variation is explained by the first principal coordinate, the second highest variation is explained by the second principal coordinate, and the third highest variation is explained by the third principal coordinate. The PCoA was displayed using R Studio and the package scatterplot3d [20]. To further explore clustering between groups, a one-way analysis of similarities (anosim) significance test was obtained by the anosim function in QIIME2 software version 2. Based on unweighted UniFrac distances, anosim is a nonparametric test to evaluate whether variation among groups is significantly larger than variation within groups, evaluating the reasonability of the division of groups. Linear discriminant analysis (LDA) combined with linear discriminant analysis effect size (LEfSe) was used to identify taxa most differing between groups and displayed via a histogram and cladogram. Phylogenetic Investigation of Communities by Reconstruction of Unobserved States (PICRUSt2) [21] was used to predict and analyse the metagenomic functions based on 16S rRNA gene sequence reads and KEGG orthology groups (KOs) using the Kyoto Encyclopedia of Genes and Genomes [22]. 

## 3. Results

**Figure 1:**
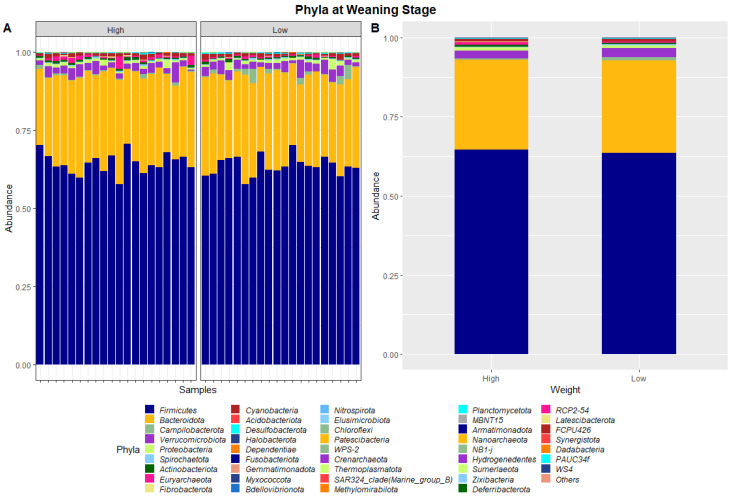
Faecal microbiota assortment at the phylum level. Bar charts representing (**A**) the relative abundance and (**B**) the average relative abundance of all bacterial ASVs taxonomically classified in each animal sampled at weaning.

The 40 samples combined from weaning and yearling stages yielded a total of 5,138,478 and 4,392,591 raw sequence reads, respectively. These raw reads underwent primer sequence removal and quality filtering, resulting in 4,672,517 (weaning) and 3,948,762 (yearling) 16S rRNA gene sequences that were subsequently analysed. The sequences were clustered into ASVs that were assigned to different levels of classification including 23 known phyla and 267 known genera at the weaning stage, and 40 known phyla and 573 known genera at yearling stage. At weaning stage, about 97% of ASVs were attributed to Firmicutes (64.1%), Bacteroidetes (28.8%), Verrucomicrobia (2.6%), Proteobacteria (0.9%), and Campilobacterota (0.8%) (Figure 1). Similarly, at yearling stage Firmicutes (57.8%), Bacteroidetes (32.3%), Proteobacteria (2.5%), Actinobacteriota (2.3%), and Cyanobacteria (0.9%) represented approximately 96% of the ASVs.

Two groups were defined based on the calculated ADG; animals with relatively higher (top 20) or relatively lower (bottom 20) ADGs were grouped as high ADG and low ADG, respectively. The average weights, means, standard deviations, and range of ADG of each of these groups are represented in Table 1. 

**Figure 2:**
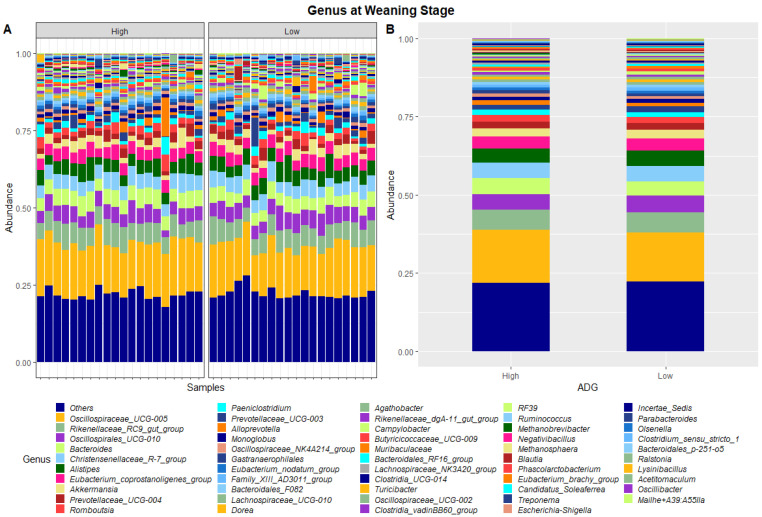
Faecal microbiota assortment at the genus level. Bar charts representing (**A**) the relative abundance and (**B**) the average relative abundance of the top 54 bacterial ASVs taxonomically classified in each animal sampled at weaning.

**Figure 3:**
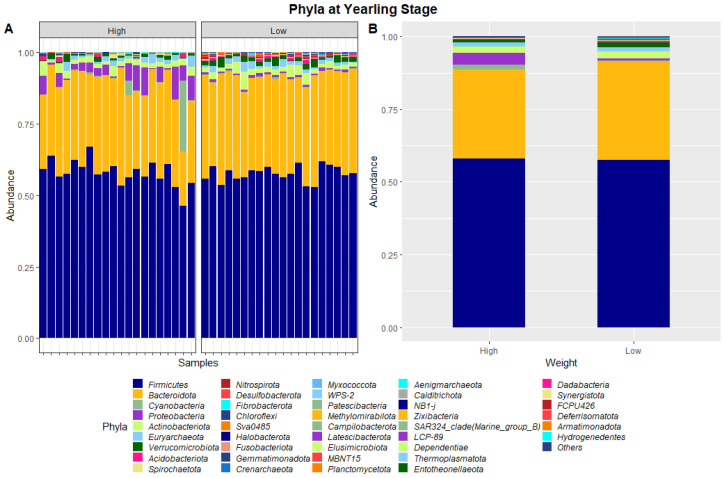
Faecal microbiota assortment at the phylum level. Bar charts representing (**A**) the relative abundance and (**B**) the average relative abundance of all bacterial ASVs taxonomically classified in each animal sampled at yearling stage.

**Figure 4:**
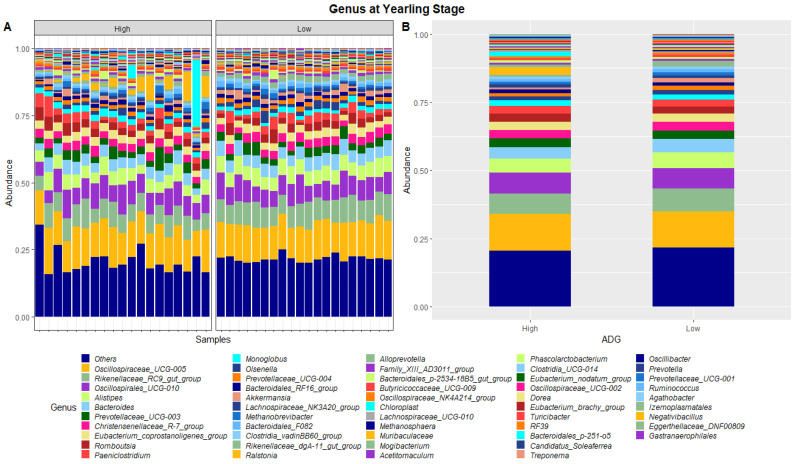
Faecal microbiota assortment at the genus level. Bar charts representing (**A**) the relative abundance and (**B**) the average relative abundance of the top 54 bacterial ASVs taxonomically classified in each animal sampled at yearling stage.

The dissimilarities in faecal microbiota profiles of high and low ADG groups at weaning and yearling stage were apparent by examining the relative abundances of ASVs assigned to different phyla and genera (Figure 1, Figure 2, Figure 3 and Figure 4). At weaning stage, the relative abundances were observed to be relatively comparable between the high and low ADG groups. However, at yearling stage, the relative abundances of specific microorganisms were found to vary significantly between the high and low ADG groups. 

Table 2 presents the phyla and genera whose relative abundances were found to differ significantly between the high and low ADG groups at both weaning and yearling stages. At weaning, the relative abundance of one phylum, Euryarchaeota, was found to significantly differ between the high and low ADG groups. At the genus level, the relative abundance of Oscillospiraceae_UCG-005 and Bacteroides were found to significantly differ between the high and low ADG groups. 

At yearling stage, eight phyla were found to have relative abundances differing significantly between the high and low ADG groups. Bacteroidota, Proteobacteria, Verrucomicrobiota, Acidobacteriota, Nitrospirota, Desulfobacterota, Chloroflexi and Sva0485 were found to significantly differ in relative abundances between the high and low ADG groups. Additionally, six genera were found to have relative abundances that significantly differed between the high and low ADG groups. Rikenellaceae_RC9_gut_group, Bacteroides, Eubacterium_coprostanoligenes_group, Monoglobus, Olsenella, and Prevotellaceae_UCG-004 were significantly different between the high and low ADG groups.

**Figure 5:**
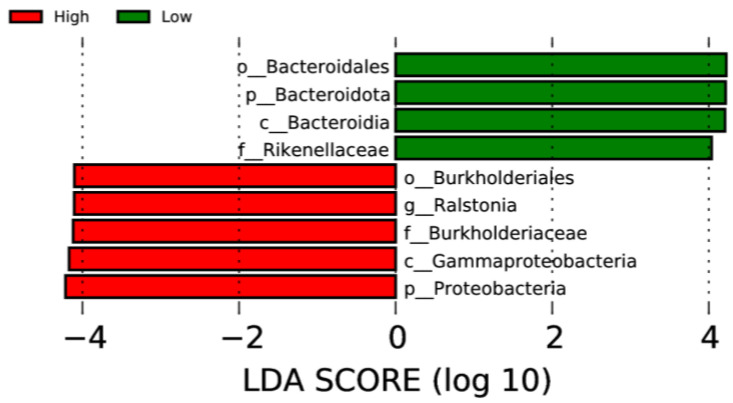
LEfSe histogram displaying enriched gut microbiota from multiple taxa levels associated with high and low ADG groups at yearling stage. Identified by linear discriminant analysis (LDA).

**Figure 6:**
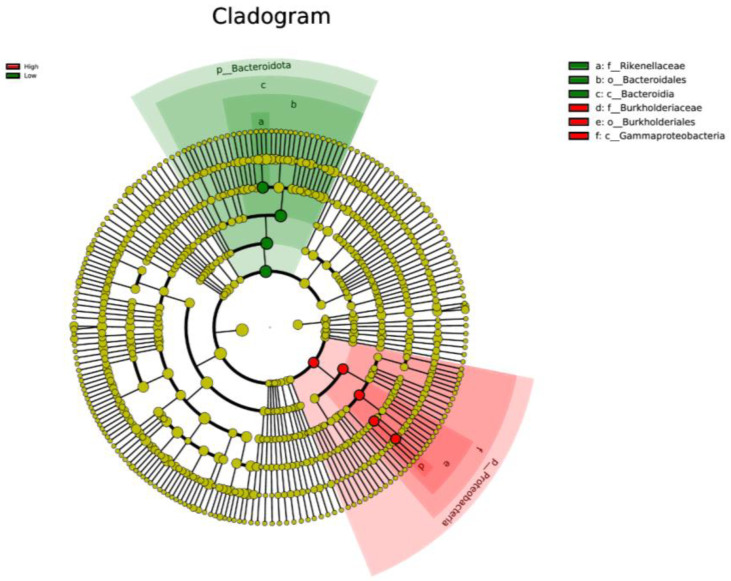
Cladogram based on LEfSe analysis displaying gut microbiota at different taxonomic levels, represented by rings (phyla in the inner most ring, species in the outer most ring, and each dot being a member within that level). Taxa are coloured by which group they are more abundant in (red = high ADG group, and green = low ADG group) with different shades representing different taxonomic levels (darkest to lightest = kingdom to species).

LEfSe analysis was used to determine taxa at different taxonomic levels that were enriched in either the high or low ADG groups at both weaning and yearling stages. No taxa were found to be enriched at weaning stage. At yearling stage, observing the histogram used to display taxa enriched in either o_Bacteroidales, p_Bacteroidota, c_Bacteroidia, or f_Rikenellaceae were overrepresented in the low ADG group (LDA score > 4), while o_Burkholderiales, g_Ralstonia, f_Burkholderiaceae, c_Gammaproteobacteria, and p_Proteobacteria were overrepresented in the high ADG group (LDA score > 4) (Figure 5). The cladogram displaying taxa enriched in each group showed similar results. No taxa were enriched in either ADG group at weaning stage. Taxa enriched at yearling stage were from the phylum Bacteroidota in the low ADG group and from Proteobacteria in the high ADG group (Figure 6). 

**Figure 7:**
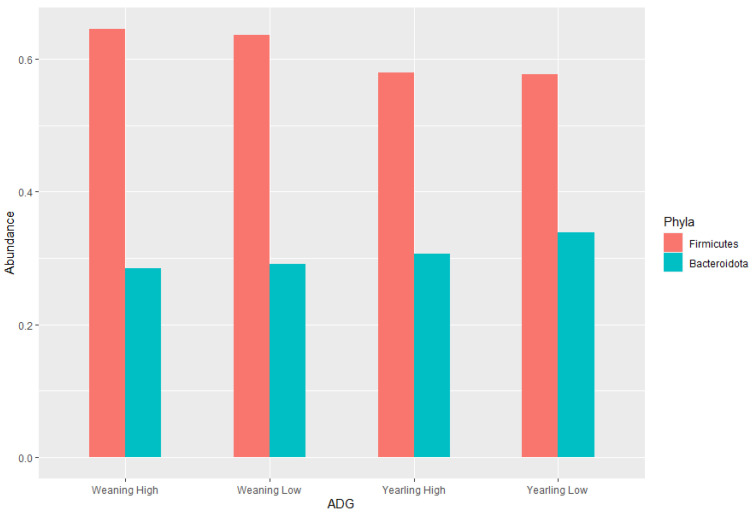
Phylum level bar charts representing the average relative abundance at weaning stage and yearling stage of the ratio of Firmicutes to Bacteroidota.

The ratio of Firmicutes to Bacteroidota within the gut has been previously described to fluctuate due to weight changes. Therefore, these two phyla were visualised separately at the weaning and yearling stages for each ADG group (Figure 7). At weaning stage, the Firmicutes-to-Bacteroidota ratio was similar between the high and low ADG groups. Furthermore, the relative abundances of these phyla were observed to have minimal differences, with Firmicutes at 0.645 in the high ADG group and 0.636 in the low ADG, and Bacteroidota at 0.285 in the high ADG with a slight increase in the low ADG group at 0.290. In comparison, at yearling stage, the difference between the Firmicutes-to-Bacteroidota ratio in the high and low groups was observed to be greater. While the abundance of Firmicutes was still relatively similar between the two ADG groups (0.579 = high ADG and 0.576 = low ADG), Bacteroidota displayed an increase in the low ADG group (0.339) compared to the high group (0.306). 

**Figure 8:**
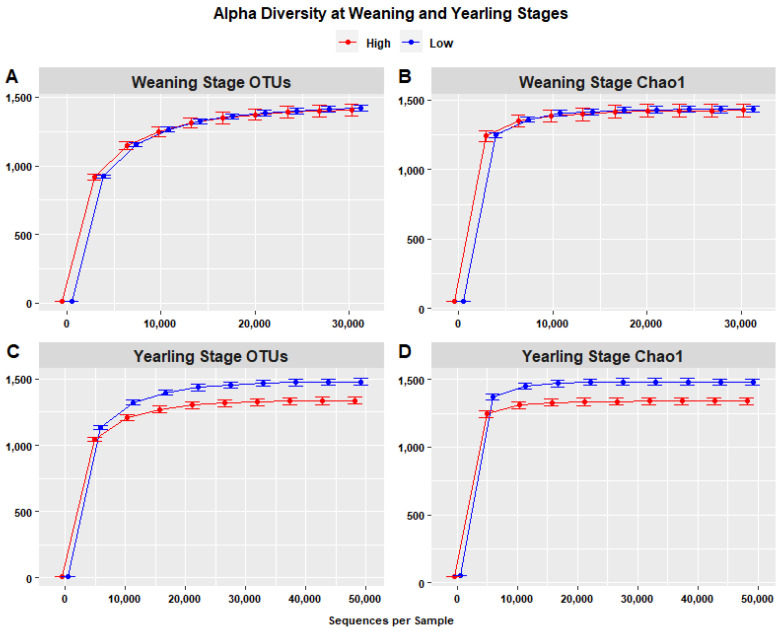
Alpha diversity (within-sample) rarefaction curves measuring species richness of the intestinal microbiota in the high (red) and low (blue) ADG groups. Observed OTUs (**A**,**C**) and Chao1 (**B**,**D**) revealing differences in species richness at both weaning and yearling stages. Error bars are staggered and representative of range.

Species richness, represented by rarefaction curves representing the number of OTUs (Figure 8A,B), indicated that species richness at the yearling stage was highest in the low ADG group (1480.4) compared to the high ADG group (1337.5) with an increase of 143.343, when considering a depth of 48,708 sequences. The difference in species richness at the weaning stage was less noticeable, with higher species richness in the low ADG group (1418.15) compared to the high ADG group (1406.4), with an increase of 6.751, when considering a depth of 30,778 sequences per sample. Krustal–Wallis pairwise tests, performed to assess the statistical significance of differences in alpha diversity, indicated that the high and low ADG groups did not significantly differ in terms of observed OTUs (*p* = 0.850), whereas at yearling stage, the two groups were significantly different (*p* = 0.001), with the low ADG group exhibiting higher alpha diversity than the high ADG group. 

Chao1 rarefaction curves were used to evaluate the sufficiency of sequencing and species richness in high and low ADG groups at both weaning and yearling stages. Chao1 is sensitive to rare species, meaning that if Chao1 is determined to have a larger sequencing depth compared to the sequencing depth of OTUs, the sequencing depth is deemed insufficient. At weaning, the Chao1 index was highest in the low ADG group (1433.038) compared to the high ADG group (1426.287) when considering a depth of 30,778 sequences/sample. At yearling, the Chao1 index in the low ADG group was highest (1483.847) compared to the high ADG group (1340.504) when considering a depth of 32,475. As the sequencing depths were consistent with the sequencing depths in the plots representing the number of OTUs, sequencing can be considered sufficient. Additionally, the rarefaction curves of both weaning and yearling stage samples attained a plateau around a sequencing depth of 21 thousand sequences (Figure 8B,D), further indicating that the sequencing depth of 30,000–50,000 sequences per sample was more than sufficient to estimate alpha diversity.

**Figure 9:**
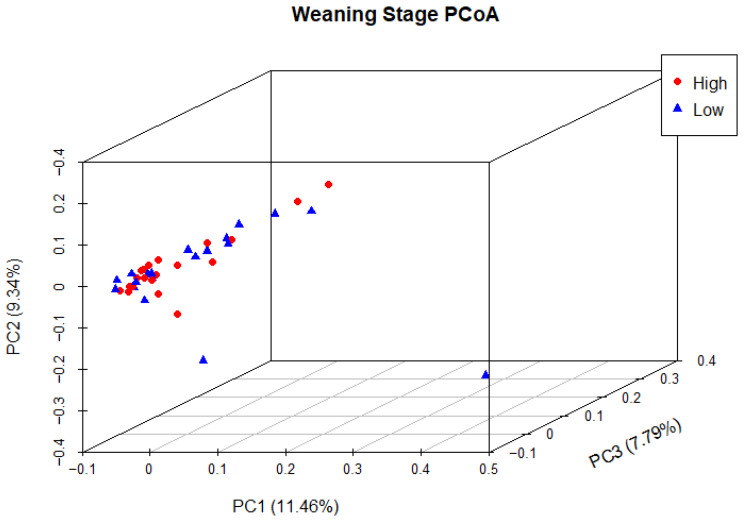
Principal coordinate analysis of the intestinal microbiota of Angus cattle at weaning stage. Plots of unweighted UniFrac distances by weight group display the beta diversity (between-sample phylogenetic similarity) for each group. Each colour represents a different weight group, either high or low, and each dot represents an individual sample. Dots that appear more closely together are more similar. The percent variation explained by the principal coordinates is indicated on the axes.

**Figure 10:**
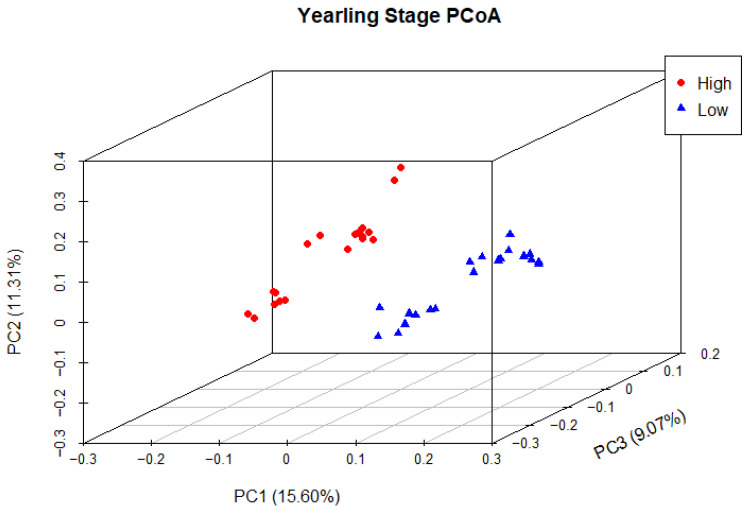
Principal coordinate analysis of the intestinal microbiota of Angus cattle at yearling stage. Plots of unweighted UniFrac distances by weight group displays the beta diversity (between-sample phylogenetic similarity) for each group. Each colour represents a different weight group, either high or low, and each dot represents an individual sample. The dots that appear more closely together are more similar. The percent variation explained by the principal coordinates is indicated on the axes.

Principal coordinate analysis (PCoA) was used to determine phylogenetic similarity between samples at both weaning and yearling stages. Unweighted UniFrac distances were used to emphasise the presence or absence of relevant ASVs (microbial species). The first three principal components were used to visualise the data, representing 28.59% and 35.98% of the overall variance in the weaning and yearling datasets, respectively. At weaning stage, samples from both the high and low ADG groups did not appear to cluster, and there was no clear separation between groups (Figure 9). Anosim analysis confirmed that there was no significant difference between the two ADG groups at weaning (*p* = 0.677). At yearling stage, samples belonging to high and low ADG groups appeared to cluster in distinctly separate groups (Figure 10). Furthermore, anosim analysis of variance confirmed that the separation between the two ADG groups was statistically different (*p* = 0.005). This indicates significant structural differences between the two ADG groups at yearling stage.

## 4. Discussion

The influence of gut microorganisms on host physiology has been well documented [5,6], with previous studies showing that gut microbiota can modulate metabolism, energy homeostasis, gut epithelial health, and digestion, all of which are factors that contribute to growth [5,6,7,8]. Additionally, studies have shown that the type and abundance of gut microorganisms can influence feed efficiency and ADG in ruminants [4,9,23]. More specifically, a higher proportion of Firmicutes to Bacteroidetes in the gut has been correlated with obesity in humans, as well as a higher milk fat composition in dairy cattle [4,9,10,24]. Given the physiological relationship between ruminal microorganisms and performance in cattle, a majority of bovine studies have focused on ruminal microorganisms [9,25,26,27]. However, there are reports that indicate that faecal microbiota profiles can be informative in terms of the health and performance of cattle [28]. Faecal microbiota largely represents hindgut microorganisms that have distinct physiological roles compared to ruminal microorganisms in terms of the health and performance of cattle. However, the collection of faecal samples is less invasive and laborious than collecting rumen samples. Therefore, if faecal microbiota profiles are found to be associated with health and performance traits, they would offer a tractable alternative to profiling ruminal microbiota on a large scale. Therefore, this study is focused on investigating the relationship between faecal microbiota and ADG in cattle. 

In the present study, the faecal microbial profiles were observed to be relatively similar between high and low ADG groups at weaning stage. Only one phylum, Euryarchaeota, was found to have relative abundances that significantly differed between the high and low ADG groups at weaning stage (*p* = 0.036). At the genus level, this trend was continued with only two genera, *Oscillospiraceae_UCG-005* and *Bacteroides*, having relative abundances that differed significantly between the high and low ADG groups at weaning stage. 

At yearling stage, the relative abundances of microbiota were observed to be visibly different between the high and low ADG groups. A MANOVA indicated eight phyla, namely, Bacteroidota, Proteobacteria, Verrucomicrobiota, Acidobacteriota, Nitrospirota, Desulfobacterota, Chloroflexi, and Sva0485, and six genera, namely, *Rikenellaceae_RC9_gut_group*, *Bacteroides*, *Eubacterium_coprostanoligenes_group*, *Monoglobus*, *Olsenella*, and *Prevotellaceae_UCG-004*, differed significantly in relative abundances between the high and low ADG groups. 

Of these, *Bacteroides* were the only taxa to significantly differ at both weaning stage and at yearling stage. *Bacteroides* are cellulolytic bacteria that exist in gut of animals and work to degrade fibre, specifically in the rumen. It was found that high-fibre diets increased the abundance of *Bacteroides* in the rumen and that this could have a flow on effect into the hindgut [29]. As this genus was found to significantly differ in abundances at both the weaning and yearling stages in the hindgut, further research maybe warranted to determine its functional relevance in the hindgut and investigate whether the observed difference in abundance physiologically contributes to high and low ADG or is the consequence of it.

The LEfSe analysis was used to determine specific taxa that were significantly enriched in either the high or low ADG groups at both the weaning and yearling stages. At weaning, no taxa were significantly enriched in either the high or low ADG groups. At yearling stage, however, four taxa were found to be significantly enriched in the low ADG group and five taxa were found to be significantly enriched in the high ADG group.

LEfSe analysis showed that Bacteroidota were significantly enriched in the low ADG group (LDA = 4). This aligns with previous observations made in this study, as Bacteroidota were found to significantly differ between the high and low ADG groups at yearling stage (*p* = 0.009). Past studies have shown that the abundance of Bacteroidota may indicate weight when viewed in conjunction with Firmicutes [9,10,24]. Specifically in ruminants, it was found that a lower abundance of Bacteroidota, when compared to Firmicutes, is correlated with high ADG. Moreover, when Bacteroidota abundances increase, ADG is found to decrease [9]. 

The LEfSe analysis found the phylum Proteobacteria to be significantly enriched in the high ADG group at yearling stage. Proteobacteria are commonly present in the gut of animals, and high abundances have previously been associated with disease and gut microbial imbalances [30]. However, a study conducted on obese children found that Proteobacteria were significantly more abundant in the obese group when compared to the undernourished group [24]. While conducted in humans, these findings do align with current observations and may suggest the abundance of Proteobacteria is relevant in relation to weight changes in both humans and cattle. However, further research is needed to determine if this phylum could be a microbial indicator for high ADG in beef cattle.

At the genus level, the LEfSe analysis displayed *Ralstonia* as being significantly enriched in the high ADG group at yearling stage. *Ralstonia* has been found to have an increased relative abundance in obese subjects with pre-diabetes and has been shown to exacerbate glucose tolerance in diet-induced obese mice [31]. Therefore, it is possible that this microorganism is functionally relevant in contributing to the high weight gain observed in the high ADG group and warrants further investigation in beef cattle.

Overall, a discernible difference was noted in the faecal microbiota profiles of cattle at weaning and yearling stages. Although microbial profiles at weaning were found to be relatively similar across both high and low ADG groups, they were markedly different at the yearling stage. These results suggest that faecal microbiota profiling at weaning may not provide an accurate indication of future growth performance of cattle. In line with this, a previous study highlighted that the period before weaning is not an optimal predictor of weight gain, as the calves are dependent on the cow. Therefore, weight gain is largely determined by maternal performance, rather than individual performance [32]. Moreover, consistent with our observations, the period before weaning is not recommended for predicting the weight gain of cattle. 

The overall trend of relatively similar faecal microbial profiles at weaning and divergent profiles at yearling stage was further validated by alpha diversity analysis. Specifically, the OTU and Chao1 plots revealed comparable species richness for both ADG groups at weaning. In comparison, species richness was significantly different between the high and low ADG groups at the yearling stage (*p* = 0.0001). This further underscores the conclusion that weaning faecal microbial profiles may not be indicative of the future growth performance of cattle.

Alpha diversity analyses revealed that species richness was reduced in the high ADG group, particularly at yearling stage, and this may be of interest due to findings from past studies. A study in cattle found that lower species richness in the rumen was strongly correlated with higher feed efficiency [25]. Specifically in the hindgut, species richness was observed to be lower in dairy cattle with greater milk production [33]. Additionally in humans, obese individuals had lower species richness in their distal gut microbial communities compared to lean individuals, and diet-induced obesity was found to be associated with a marked reduction in the overall diversity of the microbial communities [34,35,36]. While these studies are not specifically based on the hindgut of beef cattle, they have discussed underlying theories for this relationship between microbial diversity and weight gain, and these may give weight to observations made in this study. It is theorised that feed-efficient animals have a less diverse but more efficient microbial community structures in the gut [25]. Cattle with greater species richness likely have microbial communities that produce a greater array of metabolites and resource compounds that do not contribute to weight gain. However, cattle with lower species richness may have a less diverse but more specialised microbial community that can efficiently harvest energy from food and contribute to weight or production. Reports of reduced microbial diversity in animals exhibiting greater feed efficiency or ADG warrants further investigation as it could potentially serve as an indicator for growth associated traits in beef cattle [25]. 

Beta diversity analysis revealed similar trends to previous observations in this study across both weaning and yearling stages. At weaning stage, the PCoA demonstrated that samples from the high and low ADG groups were closely clustered together with no discernible separation between the two groups. This pattern indicated a high degree of between-sample phylogenetic similarity, as well as a relatively similar species composition structure between the two ADG groups. Anosim analysis based on unweighted Unifrac distances did not reveal a statistically significant difference between the two ADG groups (*p* = 0.676617), confirming that the variations between the two groups were not significantly larger than the variations within the groups.

In contrast to weaning, the PCoA at yearling stage exhibited a distinct separation between the high and low ADG groups, as evidenced by the clustering of each group. This indicated low between-group phylogenetic similarity, as well as a relatively higher degree of phylogenetic similarity within the ADG groups. Anosim analysis revealed a significant difference between the two ADG groups at the yearling stage (*p* = 0.00498), further confirming these findings.

A similar study, evaluating the faecal microbial profiles of steers with divergent feed efficiencies from weaning to slaughter, produced findings similar to this study [23]. The previous study used feed efficiency in lieu of ADG to classify animals into high and low groups, demonstrating that faecal microbiota exhibited marked changes at different life stages of the steers [23]. However, in contrast to this study, the previous study found that species richness was consistently higher in the most feed efficient steers at different life stages. While feed efficiency and ADG are different traits, it would be interesting for future studies to investigate the relationship between growth performance traits such as feed efficiency, ADG, and faecal microbiota profiles in beef cattle. 

It is important to note that the animals included in this study were all born within a 36 day window and there was no apparent substructure in terms of age in the high and low ADG groups, with both groups having animals born in overlapping periods of time. Therefore, it is unlikely that differences in faecal microbiota of yearling cattle in high and low ADG groups were driven by differences in age or the stage of growth of cattle. However, future investigations aiming to characterise the temporal changes in faecal microbiota in different growth stages may be warranted.

## 5. Conclusions

Overall, the most significant changes in microbial profiles between high and low ADG groups were observed at yearling stage. Differences in microbial profiles at yearling stage were attributed to significant differences in the relative abundance of different taxa, with a significant reduction in species richness in the high ADG group and a significant decrease in between-sample phylogenetic similarity between the two ADG groups. A MANOVA analysis indicated that eight phyla and six genera differed significantly in relative abundance between the two ADG groups, and LEfSe analysis identified nine taxa significantly enriched at yearling stage. The Firmicutes to Bacteroidota relative abundance ratio was also found to differ between yearling steers with high or low ADG. In keeping with the literature, there was a significant enrichment of Bacteroidota in the low ADG group, with a decrease in the relative abundance of Firmicutes. Overall, these results indicate that faecal microbiota profiling could potentially be used to distinguish between cattle with high and low growth performance after weaning, which could create opportunities for management-based interventions to improve the performance and profitability of beef farming enterprises. Future investigations are needed to determine if they can also be used to predict associated traits such as feed efficiency or methane emissions. Further investigation is also needed to characterise the function of these microorganisms, specifically in the hindgut, could provide novel insights that improve growth performance in beef cattle. 

## Figures and Tables

**Table 1:** Weaning weight, yearling weight, and average daily gain (ADG) of the high and low growth groups.

ADG Groups	Weaning Weight ± SD	Yearling Weight ± SD	ADG ^1^ (kg/d) with SD	Range (kg/d)
High	212.40 ± 27.06	455.40 ± 29.84	0.94 ± 0.06	1.10–0.89
Low	217.40 ± 16.66	402.10 ± 24.66	0.71 ± 0.06	0.77–0.58

^1^ ADG was calculated by weighing cattle at weaning and yearling stages and dividing the difference by the total number of days between weaning and yearling stages.

**Table 2:** Phyla and genera that significantly differed between the high and low ADG groups at both weaning and yearling stages.

**Weaning**	
Phylum	*p*-value
Euryarchaeota	0.036 *
Genus	*p*-value
*Oscillospiraceae_UCG-005*	0.034 *
*Bacteroides*	0.016 *
**Yearling**	
Phylum	*p*-value
Bacteroidota	0.009 *
Proteobacteria	0.00005 *
Verrucomicrobiota	0.003 *
Acidobacteriota	0.00002 *
Nitrospirota	0.0000000001 *
Desulfobacterota	0.000004 *
Chloroflexi	0.0002 *
Sva0485	0.000000006 *
Genus	*p*-value
*Rikenellaceae_RC9_gut_group*	0.006
*Bacteroides*	0.006 *
*Eubacterium_coprostanoligenes_group*	0.009 *
*Monoglobus*	0.0007 *
*Olsenella*	0.032 *
*Prevotellaceae_UCG-004*	0.004 *

* Indicates a significant value.

## Data Availability

The sequencing data is available via NCBI’s Sequence Read Archive (SRA) with the BioProject number PRJNA1019894.
